# Transcriptome and miRNAome analysis reveals expression profiles of platycodin biosynthesis-related genes and their potential miRNA regulators in *Platycodon grandiflorus* under high-temperature stress

**DOI:** 10.3389/fpls.2026.1820112

**Published:** 2026-05-22

**Authors:** Ling Meng, Junjie Guan, Xu Sun, Chun Wang, Pushun Luan, Fei Pan, Rongchun Han, Tao Wei, Shihai Xing

**Affiliations:** 1 College of Pharmacy, Anhui University of Chinese Medicine, Hefei, China; 2 Institute of Traditional Chinese Medicine Resources Protection and Development, Anhui Academy of Chinese Medicine, Hefei, China; 3 MOE-Anhui Joint Collaborative Innovation Center for Quality Improvement of Anhui Genuine Chinese Medicinal Materials, Hefei, China; 4 College of Integrated Chinese and Western Medicine, Anhui University of Chinese Medicine, Hefei, China; 5 The First Clinical School, Anhui University of Chinese Medicine, Hefei, China

**Keywords:** gibberellin, high-temperature stress, miRNA, MiRNA-mRNA regulatory network, platycodin, Platycodon grandiflorus, transcriptome

## Abstract

*Platycodon grandiflorus* is a widely used medicinal and edible species in Asia. With ongoing global climate warming, the rising incidence of extreme heat episodes has emerged as a major constraint on its productivity. To dissect the molecular regulatory network governing the response of *P. grandiflorus* to high-temperature stress, this study measured physiological indices and performed transcriptome sequencing (RNA-seq) on seedlings treated at 40 °C for 0, 1, 6, 12, 24, and 48 h. This was combined with microRNA (miRNA) sequencing data from the 0 h and 48 h time points for an integrated multi-omics analysis. Under high-temperature (HT) stress, the activities of superoxide dismutase (SOD), peroxidase (POD), and ascorbate peroxidase (APX) were markedly elevated, whereas malondialdehyde (MDA) and hydrogen peroxide (H_2_O_2_) levels exhibited a transient increase followed by a decline. A comparison between the control and five HT treatment groups identified 1019 common significantly differentially expressed genes. miRNA sequencing identified 62 HT-responsive differentially expressed miRNAs targeting 157 predicted genes, and the integrated analysis further screened 32 differentially expressed target genes. Based on this, the study focused on elucidating the expression profiles of structural enzyme genes (*BASs, CYPs, UGTs, BGLUs*) in the platycodin biosynthesis pathway. It was found that upstream genes involved in terpenoid backbone synthesis were generally down-regulated, whereas downstream genes related to oxidative modification and glycosylation dynamics exhibited differential response patterns. Integrated analysis further revealed that ent-kaurene synthase (KS), the major rate-limiting enzyme in the gibberellin (GA) biosynthesis pathway, is potentially co-targeted by *pgy-miR408-3p-3* and *pgy-miR408d* at the post-transcriptional level and exhibits significantly up-regulated expression. Conversely, *BGLU11*, a β-glucosidase gene involved in saponin deglycosylation, was negatively regulated by *pgy-miR395b-3* and exhibited significantly down-regulated expression. These results suggest that under HT stress, *P. grandiflorus* employs a complex miRNA-mRNA regulatory network to mediate a potential redistribution of terpenoid metabolic flux between the saponin and gibberellin pathways, while dynamically modifying saponin structures to maintain metabolic homeostasis. This study constructs a transcriptional and post-transcriptional regulatory framework for the response of *P. grandiflorus* to HT stress, providing key genetic resources and molecular clues for in-depth analysis of its thermotolerance mechanisms and for stress-resistance breeding.

## 1 Introduction

*Platycodon grandiflorus* is a perennial herb of the Campanulaceae with well-recognized nutritional and medicinal importance. Its roots are rich in diverse metabolites, notably triterpenoid saponins, polysaccharides, and phytosterols. Of these constituents, oleanane-type triterpenoid saponins are widely regarded as the major pharmacologically active molecules, contributing to reported antitumor, anti-inflammatory, antihyperlipidemic, and hepatoprotective effects (Kim et al., 1995; Lee et al., 2004; Kim et al., 2008; Lee et al., 2008; Kim et al., 2013). *P. grandiflorus* is widely distributed and cultivated across East Asia. However, with the intensification of global warming and the rising frequency of extreme temperature events, the cultivation industry of *P. grandiflorus* is facing a severe threat.

Plant heat stress is defined as a condition in which prolonged exposure to temperatures exceeding a species-specific threshold leads to physiological dysfunction (Xalxo et al., 2020). Plants cope with high-temperature (HT) stress by triggering coordinated physiological and biochemical adjustments. Heat stress often leads to the overaccumulation of reactive oxygen species (ROS), thereby disrupting membrane integrity and impairing protein function. To mitigate oxidative injury, plants promptly induce antioxidant defense pathways that detoxify and remove excess ROS (Almeselmani et al., 2006). At the molecular level, these physiological and biochemical alterations are attributed to the induction of a broad spectrum of proteins, encompassing both functional and regulatory proteins, as well as non-coding RNAs. This process is regulated by the concerted HT-induced expression of multiple genes (Huang et al., 2022). Although the core regulatory networks governing heat stress response, particularly the well-characterized pathways mediated by heat shock transcription factors (HSFs) in model species like *Arabidopsis thaliana* (Driedonks et al., 2015; Andrási et al., 2021), the molecular determinants of HT stress acclimation in *P. grandiflorus* have yet to be fully elucidated.

The seedling stage is widely regarded as one of the most sensitive windows to environmental stress and a critical period for the establishment and consolidation of stress resistance (Gommers and Monte, 2018). Stress responses at this stage are often more rapid and pronounced, and the underlying molecular regulatory mechanisms likely represent evolutionally conserved, fundamental defense programs. Consequently, in fundamental plant stress biology research, leveraging the high sensitivity of the seedling stage to dissect core stress-responsive pathways has become a common and effective strategy for uncovering the essence of plant stress tolerance. For instance, in *Medicago truncatula*, multi-omics analyses using seedlings successfully revealed a widespread translation regulatory network independent of the transcriptional level under salt stress, highlighting the advantage of the seedling model in uncovering sophisticated molecular mechanisms (An et al., 2023). Similarly, drought hardening treatment of potato (*Solanum tuberosum* L.) seedlings can induce a series of physiological, biochemical, and morphological adaptive changes that enhance drought resistance, providing direct evidence for early-stage stress management in crops (Zhang et al., 2018).

With the rapid advancement of genomic technologies, transcriptome sequencing (RNA-seq) has emerged as a highly effective tool for deciphering complex biological processes in plants. It has been widely employed in plant stress resistance research to screen differentially expressed genes and elucidate their functional mechanisms (Gill and Dhillon, 2022). Through differential transcriptomic analysis, numerous key genes involved in stress responses have been successfully identified. For instance, in rice (*Oryza sativa*) under low-temperature stress, *OsCATC* and *Os03g0701200* were identified and validated as candidate target genes for improving cold tolerance (Zhong et al., 2024). In salt stress studies, analyzing the differential transcriptomic profiles and alternative splicing patterns of barley (*Hordeum vulgare*) and rice (*O. sativa*) revealed the molecular basis underlying the superior salt tolerance of barley (Fu et al., 2019). Furthermore, RNA-seq has been extensively applied in research on plant responses to drought (Swenson et al., 2017; Murtaza et al., 2025), high-temperature stress (Ma et al., 2024; Luo et al., 2025), and biotic stress (Guo et al., 2023). microRNAs are endogenous, short noncoding RNA molecules that fine-tune gene expression primarily through post-transcriptional repression (Rubio-Somoza and Weigel, 2011; Iwakawa and Tomari, 2015). Since their initial discovery in *Caenorhabditis elegans* in 1993 (Wightman et al., 1993), the functions of miRNAs in gene regulatory networks of both animals and plants have become a research focus. Recent studies have demonstrated that miRNAs are extensively involved in various plant physiological processes, including growth and development, hormone signal transduction, and stress responses (Liu et al., 2017). They can respond rapidly to stress cues and promote stress tolerance by fine-tuning the expression of downstream stress-responsive genes. For example, in banana, the *miR156c-MaSPL4* module mediates the low-temperature stress response by regulating the *miR528-MaPPO* module and other pathways (Kong et al., 2025). In wheat (*Triticum aestivum*), *tae-miR9668* negatively regulates *TaRPM1-1A*, whose protein product interacts with the TaSnRK1.3-D protein to participate in salt tolerance regulation (He et al., 2025).

Based on this background, the present study aimed to elucidate the molecular regulatory network underlying the high-temperature stress response in the medicinal plant *P. grandiflorus* by integrating physiological, transcriptomic, and miRNAomic analyses. We conducted a time-course experiment using HT-treated seedlings to systematically evaluate antioxidant enzyme activities and oxidative damage indicators. In parallel, we generated comprehensive expression profiles of differentially expressed genes (DEGs) and miRNAs (DEMs). Integrative multi-omics analysis enabled us to propose, for the first time in this species, a preliminary regulatory framework for platycodin metabolism under HT stress, spanning transcriptional and post-transcriptional regulation. We further identified potential crosstalk with other pathways, including gibberellin biosynthesis. Collectively, these findings provide valuable multi-omics resources and highlight key candidate genes for elucidating heat tolerance mechanisms in *P. grandiflorus*.

## 2 Materials and methods

### 2.1 Plant materials and growth conditions

Mature, non-dehiscent capsules of *P. grandiflorus* were collected, and the seeds were subsequently sown in seedling trays filled with nutrient soil. The seedlings were cultivated in a programmable growth chamber (Lvbo, Hangzhou, China) under strictly controlled environmental conditions (12 h light/12 h dark photoperiod, 25 °C) to promote uniform growth. Seed germination occurred at approximately 20 days after sowing. When the seedlings developed to a height of 10–15 cm, plants with comparable growth status were selected and used for subsequent experiments. For high-temperature treatment, the seedlings were moved to another identical growth chamber adjusted to 40 °C while maintaining the same photoperiod regime (12 h light/12 h dark). Whole seedlings were sampled at 0, 1, 6, 12, 24, and 48 h after the onset of HT exposure for transcriptome sequencing and determination of physiologzical indices. For miRNA profiling, samples were collected specifically at 0 h and 48 h following HT treatment. Each sampling time point was defined as an independent group, and three biological replicates were prepared for every group. All harvested samples were immediately snap-frozen in liquid nitrogen and then stored at −80 °C until subsequent analyses.

### 2.2 Determination of physiological parameters

The activities of antioxidant enzymes and the concentrations of malondialdehyde (MDA) and hydrogen peroxide (H_2_O_2_) were quantified for each experimental group. These determinations were carried out in accordance with the manufacturer’s standard procedures using commercial assay kits (Boxbio, Beijing, China): Superoxide Dismutase Activity Assay Kit, Peroxidase Activity Assay Kit, Ascorbate Peroxidase Activity Assay Kit, Malondialdehyde Content Assay Kit, and Hydrogen Peroxide Content Assay Kit.

### 2.3 cDNA library construction and transcriptome sequencing

Three *P. grandiflorus* seedlings were harvested at each sampling point and combined to constitute one biological replicate, yielding 18 biological samples in total. Total RNA was then isolated by ethanol precipitation, after which the CTAB-PBIOZOL method was applied for extraction. RNA quantity and integrity were evaluated using a Qubit 4.0 Fluorometer (Thermo Fisher Scientific, Waltham, USA) together with a Qsep400 Bioanalyzer (Bioptic Inc., Hsinchu, China). For RNA-seq library preparation, poly(A) mRNA was captured with Oligo(dT) magnetic beads and subsequently fragmented. Random hexamer primers were used to initiate first-strand cDNA synthesis, followed by strand-specific second-strand synthesis to generate double-stranded cDNA. The resulting cDNA was subjected sequentially to end repair, dA-tailing, and ligation of sequencing adapters. Libraries with insert sizes of 250–350 bp were size-selected via bead-based purification, amplified by PCR, and purified once more. Finally, the library concentration and fragment size distribution were re-checked using the Qubit 4.0 and Qsep400 systems. Qualified libraries were pooled at appropriate ratios and sequenced on an Illumina platform to generate 150 bp paired-end reads.

### 2.4 Data quality control and identification of DEGs

Raw sequencing data were first subjected to quality control with fastp (Chen et al., 2018) to trim adapter sequences and remove low-quality reads. For paired-end data, read pairs were excluded when either mate contained > 10% ambiguous nucleotides (N content) or when > 50% of the bases showed a Phred quality score (Q) ≤ 20. All downstream analyses were carried out using the remaining high-quality clean reads. An index was generated with HISAT2 (Kim et al., 2015), and the clean reads were then mapped to the *P. grandiflorus* reference genome. This genome is available from the Genome Warehouse (GWH) of the National Genomics Data Center (https://ngdc.cncb.ac.cn/gwh) under BioProject accession number PRJCA003843 and assembly accession number GWHARYT00000000 (Jia et al., 2022). Gene-level read counts were summarized using featureCounts (Liao et al., 2014). Subsequently, Fragments Per Kilobase of transcript per Million mapped reads (FPKM) were computed for each gene by normalizing mapped fragments to transcript length and library size. Differential expression between experimental groups was assessed with DESeq2 (Love et al., 2014; Varet et al., 2016). To account for multiple testing, raw P-values were corrected using the Benjamini-Hochberg procedure to control the false discovery rate (FDR). Genes meeting the thresholds of |log_2_Fold Change| ≥ 1 and an FDR < 0.05 were defined as significantly differentially expressed.

### 2.5 sRNA library construction and miRNA sequencing

Seedlings of *P. grandiflorus* collected at 0 h and 48 h were used for small RNA (sRNA) library preparation and sequencing. Total RNA was isolated from these samples, and RNA integrity was subsequently evaluated using a Bioanalyzer 2100 system (Agilent, Santa Clara, USA). Small RNA libraries were prepared in accordance with the manufacturer’s instructions provided with the TruSeq Small RNA Sample Prep Kits (Illumina, San Diego, USA). After quality qualification, the resulting libraries were sequenced on an Illumina HiSeq 2500 platform (Illumina, San Diego, USA), generating single-end 50 bp (SE50) reads.

### 2.6 Identification of miRNAs and prediction of their target genes

Raw sequencing reads were first processed with fastp (Chen et al., 2018) for adapter trimming and quality filtering, thereby generating high-quality clean datasets. The resulting clean reads were subsequently mapped to the aforementioned reference genome (Jia et al., 2022). using bowtie (Langmead, 2010). To focus on canonical miRNA-sized molecules, small RNA sequences were further screened by length, and only reads of 21–24 nucleotides were retained for downstream analyses. Known miRNAs were identified by aligning the filtered reads to the miRBase database (Kozomara et al., 2019). In parallel, other classes of non-coding RNAs were annotated through comparison with the Rfam database (Kalvari et al., 2021). Putative novel miRNAs were then inferred based on the presence of typical miRNA precursor hairpin structures.

For each sample, the abundance of both known and novel predicted miRNAs was quantified and normalized as Transcripts Per Million (TPM). Differential expression analysis between groups was carried out using DESeq2 (Love et al., 2014; Varet et al., 2016), and miRNAs meeting the criteria of |log_2_Fold Change| ≥ 1 and p-value < 0.05 were defined as differentially expressed. Potential target genes of the differentially expressed miRNAs were predicted with targetfinder (The targetFinder prediction score ≤ 4.0) (Bo and Wang, 2005). The resulting candidate targets were subjected to Gene Ontology (GO) enrichment analysis and Kyoto Encyclopedia of Genes and Genomes (KEGG) pathway annotation to explore their putative biological functions and associated regulatory pathways. Finally, the miRNA target set was integrated with the differentially expressed genes derived from the transcriptome analysis, and overlapping genes were extracted to highlight key candidate targets that may contribute to the response to the target stress.

### 2.7 Quantitative real-time polymerase chain reaction validation

To verify the accuracy of the transcriptome and miRNA sequencing data, qRT-PCR analysis was performed on selected differentially expressed genes and miRNA-mRNA regulatory pairs. Total RNA was extracted using TRIzol reagent, and reverse transcription to cDNA was carried out using the Evo M-MLV Reverse Transcription Premix (Accurate Biology, Changsha, China). For mRNA detection, amplification was performed using the SYBR Green Premix Pro Taq HS qPCR Kit (Accurate Biology) on a LightCycler 480 (Roche, Mannheim, Germany). For miRNA detection, stem-loop reverse transcription was employed, followed by quantification using the 2 × Universal Blue SYBR Green qPCR Master Mix (Servicebio, Wuhan, China). U6 snRNA was used as the internal reference gene for miRNAs, and 18S rRNA was used as the internal reference gene for mRNAs. The primer sequences are listed in **Supplementary Table 1**. Each sample was analyzed in triplicate, and the relative expression levels were calculated using the 2^^−ΔΔCt^ method.

### 2.8 Determination of platycodin D and gibberellin GA_3_ contents

Lyophilized powders of *P. grandiflorus* seedlings collected at 0 h and 48 h after treatment were prepared. 0.5 g of each powder was weighed, mixed with 10 mL of 70% methanol, and subjected to ultrasonic extraction for 30 min. After centrifugation, the supernatant was collected and concentrated to 1 mL, then filtered through a 0.22 µm microporous membrane prior to UPLC-ELSD analysis. The analysis was performed on an Agilent 1290 Infinity II LC system (Agilent Technologies, Waldbronn, Germany) following a previously reported method (Kwon et al., 2017). The chromatographic separation was carried out on an Agilent ZORBAX Eclipse Plus C18 column (2.1 × 100 mm, 1.8 µm). The mobile phase consisted of acetonitrile (A) and 0.1% formic acid in water (B) under a gradient elution program: from 0 min to 20 min, A increased linearly from 24% to 31%. The flow rate was 0.3 mL/min, the column temperature was maintained at 35 °C, and the injection volume was 5 µL. Quantification of GA_3_ was performed by Nanjing Ruiyuan Biotechnology Co., Ltd. using HPLC-MS.

## 3 Results

### 3.1 Physiological response of *P. grandiflorus* to high-temperature stress

**Figure 1:**
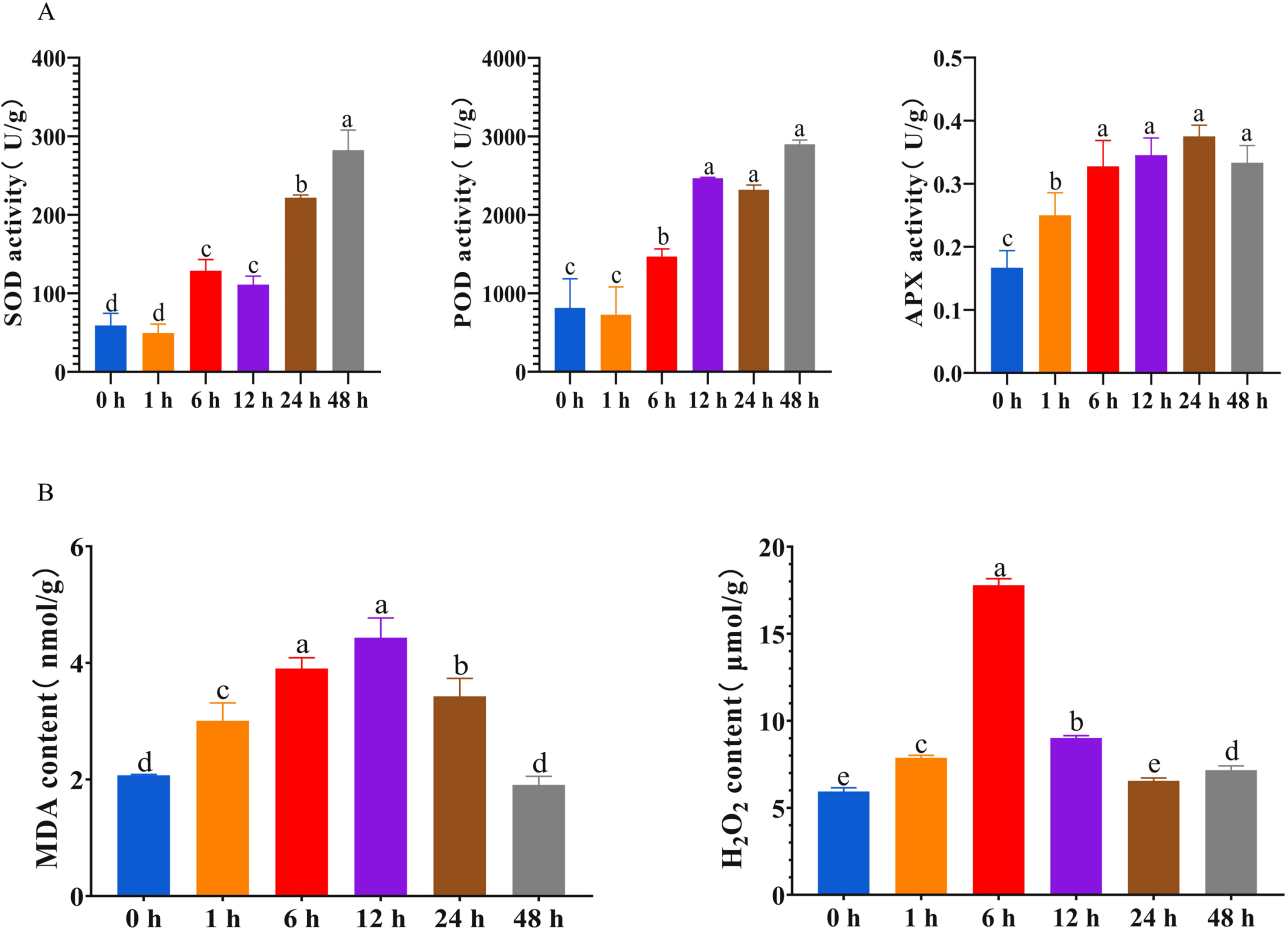
Physiological alterations in *P. grandiflorus* seedlings under high-temperature (HT) stress. **(A)** Variation in antioxidant enzyme activities. **(B)** Variation in malondialdehyde (MDA) and hydrogen peroxide (H_2_O_2_) contents. Different letters denote statistically significant differences (P < 0.05).

After exposure to 40 °C for 48 hours, the antioxidant enzyme activities in *P. grandiflorus* exhibited significant changes. The activities of superoxide dismutase (SOD), peroxidase (POD), and ascorbate peroxidase (APX) all showed an increasing trend, with the magnitudes of change in POD and APX activities decreasing after 12 h and 6 h, respectively (**Figure 1A**). Concurrently, the contents of MDA and H_2_O_2_ were measured. These two parameters are commonly used as indicators of the degree of cellular damage in plants (Montero-Palmero et al., 2014; Černý et al., 2018). The results revealed that both MDA and H_2_O_2_ contents initially increased and subsequently decreased, MDA content peaked at 12 h and then declined, while H_2_O_2_ content peaked at 6 h and then decreased (**Figure 1B**). These findings indicate that the early phase of high-temperature stress (prior to 6 h or 12 h) is characterized by a rapid oxidative stress response, whereas in the later phase, antioxidant enzyme activities tend to stabilize and the contents of MDA and H_2_O_2_ decrease, suggesting that *P. grandiflorus* may enter a new metabolic homeostasis or an adaptive adjustment stage.

### 3.2 Transcriptome sequencing analysis of the transcriptional profile in response to high-temperature stress

**Figure 2:**
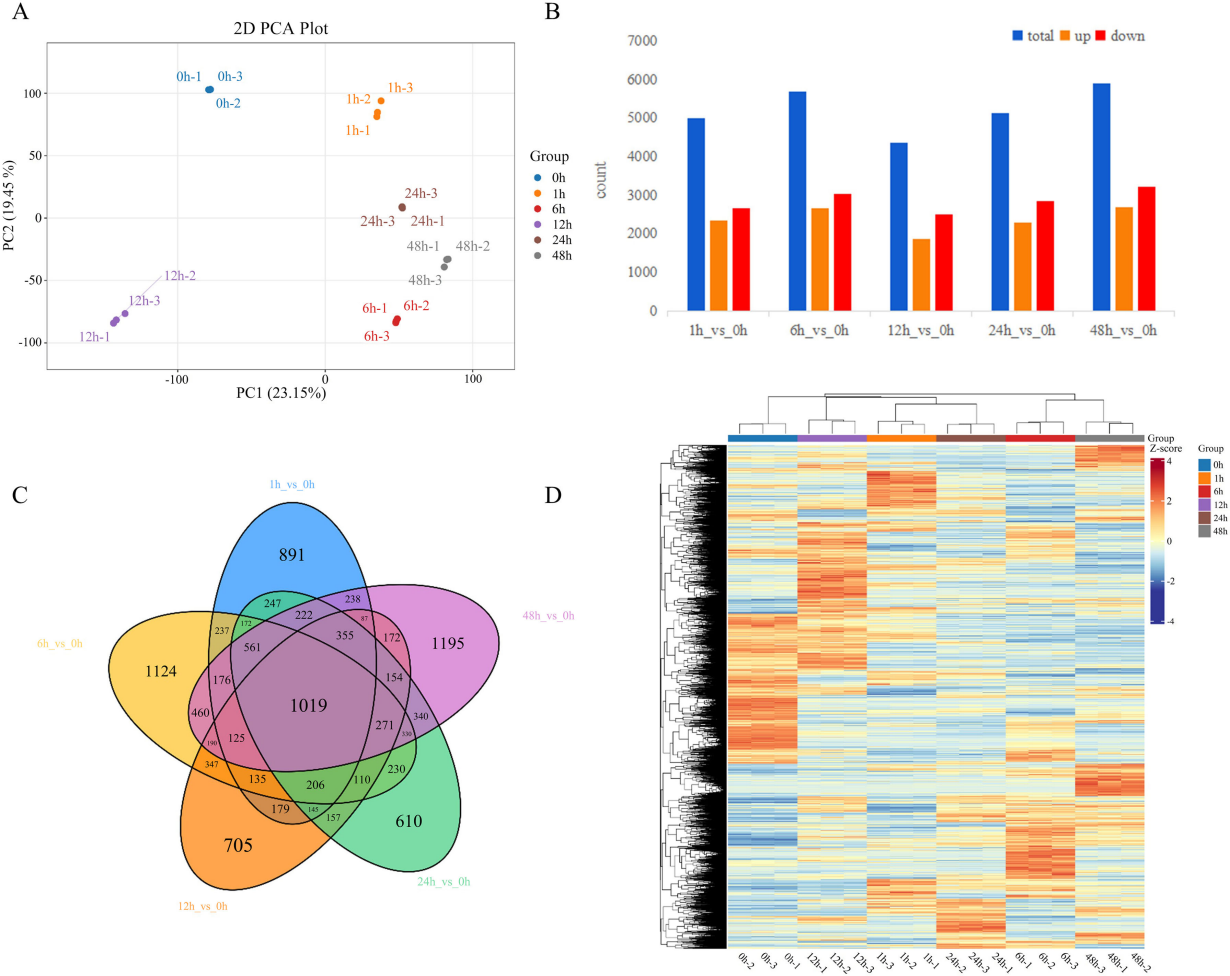
Overview of differentially expressed genes (DEGs) in response to HT stress. **(A)** Principal component analysis (PCA) performed on samples from the different groups. **(B)** Number of DEGs responsive to HT stress (|log_2_Fold Change| ≥ 1 and FDR < 0.05). **(C)** Venn diagram illustrating shared and unique DEGs among treatments. **(D)** Heatmap showing expression-pattern clustering of DEGs across the different treatments.

In this study, transcriptome sequencing was conducted to characterize the transcriptional landscape of *P. grandiflorus* under high-temperature stress. Illumina deep sequencing was applied to 18 libraries derived from six experimental groups, with three biological replicates per group, and yielded 123 Gb of high-quality clean data in total. For every individual sample, no less than 5 Gb of clean reads were obtained, and the proportion of bases with Q30 scores reached ≥ 95%, indicating robust sequencing quality. Clean reads were mapped to the *P. grandiflorus* reference genome (Jia et al., 2022), and the overall mapping rate exceeded 80% across samples. Principal component analysis (PCA) further supported the reliability of the dataset, as biological replicates within the same group clustered tightly, while distinct groups were clearly separated in the ordination space (**Figure 2A**).

Differentially expressed genes were screened using the criteria of |log_2_Fold Change| ≥ 1 combined with a false discovery rate (FDR) < 0.05. Compared to the control group (0 h), each high-temperature treatment group exhibited between 4,300 and 5,900 DEGs. Notably, the 48 h treatment group yielded the highest number, with a total of 5,895 DEGs identified, comprising 2,685 up-regulated and 3,210 down-regulated genes. Across all treatment groups, the number of down-regulated genes consistently exceeded that of up-regulated genes (**Figure 2B**). A Venn diagram illustrates the overlap among these DEGs (**Figure 2C**). A total of 1019 genes were significantly differentially expressed in all five comparison groups, forming a core set of consistently responsive transcripts to 40 °C HT stress in *P. grandiflorus*. A heatmap displays the expression profiles of all identified DEGs (**Figure 2D**).

### 3.3 GO and KEGG enrichment analyses of DEGs

**Figure 3:**
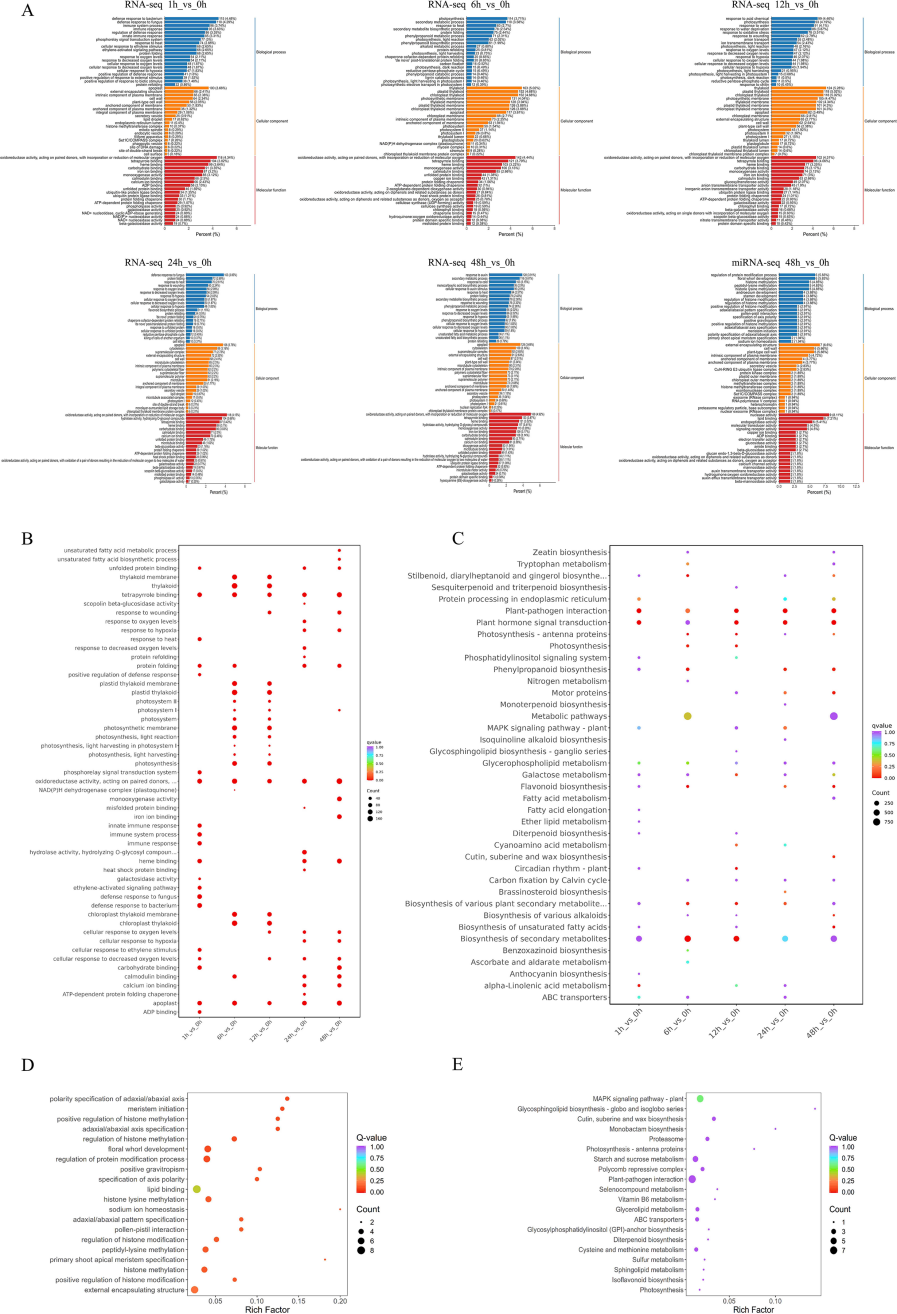
GO and KEGG enrichment analyses. **(A)** Bar graph showing the number of enriched GO terms for DEGs and target genes of differentially expressed miRNAs (DEMs). **(B)** Scatter plot of GO enrichment analysis for DEGs. **(C)** Scatter plot of KEGG pathway enrichment analysis for DEGs. **(D)** Scatter plot of GO enrichment analysis for DEM target genes. **(E)** Scatter plot of KEGG pathway enrichment analysis for DEM target genes.

To elucidate the functional implications of differentially expressed genes in response to high-temperature stress, enrichment analyses were performed using GO and KEGG. For GO analysis, the DEGs from each of the five pairwise comparisons were examined, and the top 20 most significantly enriched GO terms (smallest q-values) across the three GO categories were identified (**Figure 3A**). The results indicated that within the Biological Process category, the gene functions most significantly associated with high-temperature stress included secondary metabolic process (GO:0019748), response to heat (GO:0009408), and protein folding (GO:0006457). In the Cellular Component category, the most significantly enriched GO terms were apoplast (GO:0048046) and thylakoid (GO:0009579). In the Molecular Function category, the most enriched GO terms were oxidoreductase activity, acting on paired donors, with incorporation or reduction of molecular oxygen (GO:0016705) and monooxygenase activity (GO:0004497) (**Figure 3B**). KEGG pathway enrichment analysis was performed on the differentially expressed genes from each treatment group. The results revealed several significantly enriched pathways, including metabolic pathways (ko01100), biosynthesis of secondary metabolites (ko01110), plant-pathogen interaction (ko04626), plant hormone signal transduction (ko04075), MAPK signaling pathway-plant (ko04016), and motor proteins (ko04814) (**Figure 3C**). Detailed results of the GO and KEGG analyses for each group are provided in **Supplementary Table 2**.

### 3.4 miRNA sequencing analysis of the miRNA profile in response to high-temperature stress

**Figure 4:**
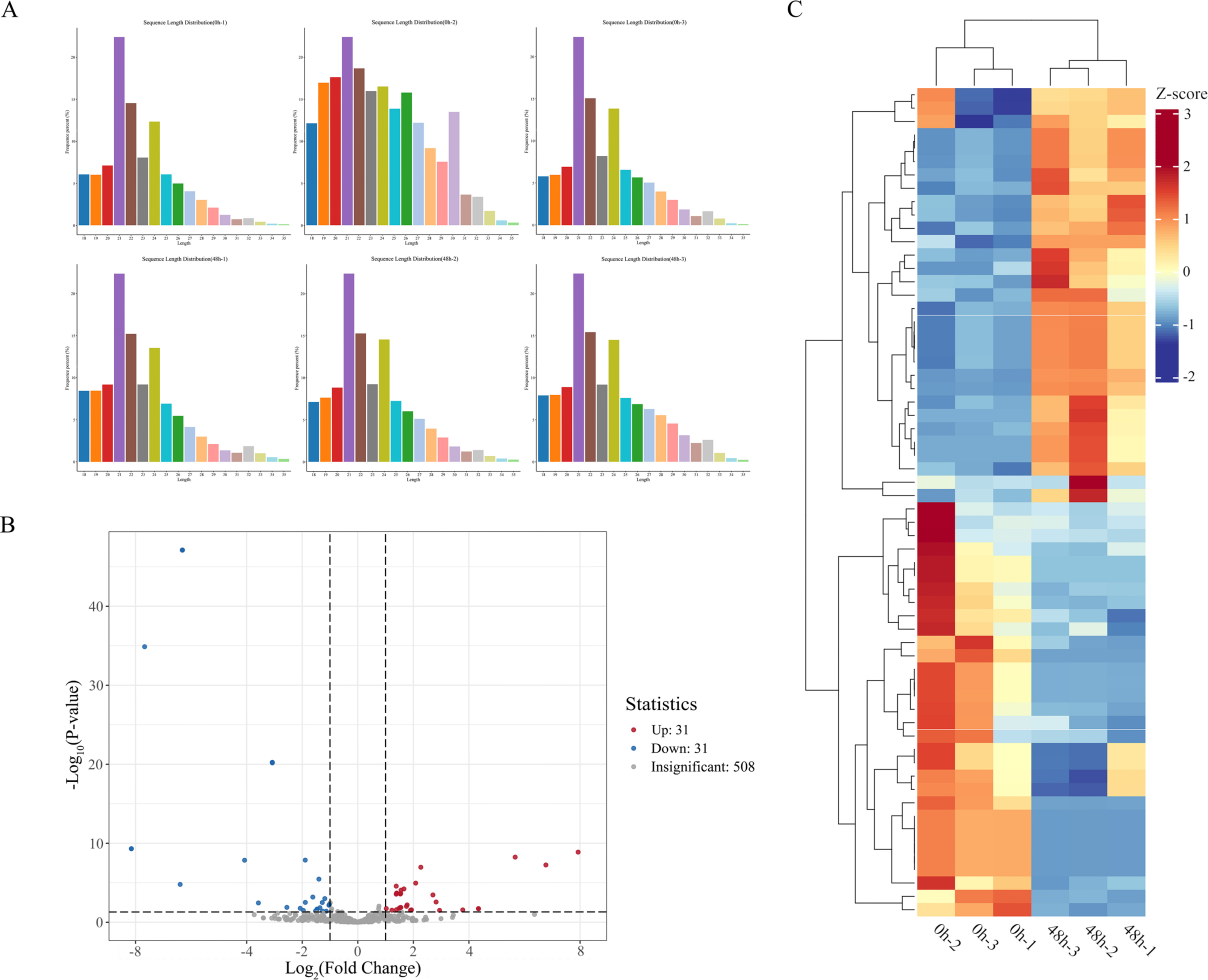
Overview of DEMs in response to HT stress. **(A)** Length distribution of small RNAs (sRNAs) from different samples. **(B)** Volcano plot of DEMs responsive to HT stress. **(C)** Heatmap of expression clustering for DEMs responsive to HT stress (|log_2_Fold Change| ≥ 1 and p-value < 0.05).

Based on the aforementioned trends in physiological indices and the overall characteristics of transcriptomic changes, the 48 h time point exhibited the highest number of differentially expressed genes (**Figure 2B**) and the most abundant enrichment of metabolism-related pathways (**Figure 3A**), indicating that transcriptomic reprogramming at this time point was the most drastic and the changes in the metabolic regulatory network were the most comprehensive compared with the other time points. Therefore, in the present study, the groups at 0 h and 48 h were selected for miRNA analysis. The Result of miRNA sequencing results showed between10.2 to 12.7 million raw reads. After removing adapters, low-quality reads, and other irrelevant information, between 7.4 to 9.9 million clean reads were obtained. A total of 629 miRNAs were identified across all samples, including 542 known miRNAs and 87 novel predicted miRNAs. In this study, known miRNAs were named using the species-specific prefix “pgy” for *P. grandiflorus*, while novel predicted miRNAs were designated with the locus identifier “GWHARYT” derived from the reference genome as their prefix. For traceability and comparison, the original official species names of these miRNAs from miRBase are also retained in the supplementary materials; details are provided in **Supplementary Table 3**. Length distribution statistics showed that the miRNAs were predominantly enriched in sequences ranging from 21 to 24 nt, with the 21-nt fragment being the most abundant (**Figure 4A**). This distribution aligns with previous reports (Ma and Hu, 2023).

Differentially expressed miRNAs were screened using thresholds of |log_2_Fold Change| ≥ 1 and p-value < 0.05. A total of 62 miRNAs were found to be differentially expressed in response to high-temperature stress. Among these HT-responsive miRNAs, 31 were up-regulated and 31 were down-regulated (**Figures 4B, C**).

### 3.5 GO and KEGG enrichment analyses of potential target genes of DEMs

To elucidate the biological roles of the high-temperature-responsive miRNAs, we predicted target genes (The targetFinder prediction score ≤ 4.0) (Bo and Wang, 2005) for the significantly differentially expressed miRNAs, identifying 157 potential targets for the 62 HT-responsive ones.

To further elucidate the potential biological roles of the predicted target genes in the response to HT stress, GO and KEGG enrichment analyses were conducted. GO enrichment results revealed that within the Biological Process category, the most significant GO terms were floral whorl development (GO:0048438), regulation of protein modification process (GO:0031399), histone lysine methylation (GO:0034968), regulation of histone modification (GO:0031056), and regulation of histone methylation (GO:0031060). In the enriched Cellular Component category, the most overrepresented terms were external encapsulating structure (GO:0030312), plant-type cell wall (GO:0009505), and cell wall (GO:0005618). Within the Molecular Function category, the most significant GO terms were nuclease activity (GO:0004518), lipid binding (GO:0008289), signaling receptor activity (GO:0038023), and beta-glucosidase activity (GO:0008422) (**Figures 3A, D**). KEGG enrichment analysis showed that these genes were significantly enriched in pathways such as Plant-pathogen interaction (ko04626) and MAPK signaling pathway-plant (ko04016) (**Figure 3E**). Therefore, it is hypothesized that these differentially expressed genes may constitute important components of the transcriptional regulatory network mediating *P. grandiflorus* response to high-temperature stress.

### 3.6 Integrated analysis of transcriptome and miRNAome

An integrated analysis of differentially expressed miRNAs and their target genes was conducted to explore the potential role of miRNA-mRNA regulatory networks in the biological processes of *P. grandiflorus* under high-temperature stress. By combining the transcriptome and miRNAome data from the 0 h and 48 h groups showed that 32 out of the 157 predicted target genes were themselves differentially expressed after HT treatment. These 32 differentially expressed target genes corresponded to 58 miRNA-mRNA regulatory pairs. In plants, miRNAs mainly negatively regulate gene expression by mediating of target mRNAs degradation (Iwakawa and Tomari, 2015), thereby inhibiting their translation. Among these 58 pairs, 12 pairs exhibited an up-regulated miRNA coupled with a down-regulated mRNA, while 15 pairs showed a down-regulated miRNA coupled with an up-regulated mRNA. In these miRNA-mRNA interactions, individual target genes may be co-regulated by multiple miRNAs, whereas a given miRNA can simultaneously modulate multiple target transcripts. For instance, the decreased expression of *GWHARYT00000233_0-5p* was associated with increased expression of its target genes PGRA_12150, PGRA_02490, PGRA_20750, and PGRA_08788. Conversely, the expression of the target gene PGRA_04019 decreased in response to the increased expression of the differentially expressed miRNAs *pgy-miR408-1*, *pgy-miR408d*, *pgy-miR408-3p-2*, *pgy-miR408-2*, *pgy-miR408-3p-1*, and *pgy-miR408-3p-3*.

### 3.7 Validation by quantitative real-time polymerase chain reaction

**Figure 5:**
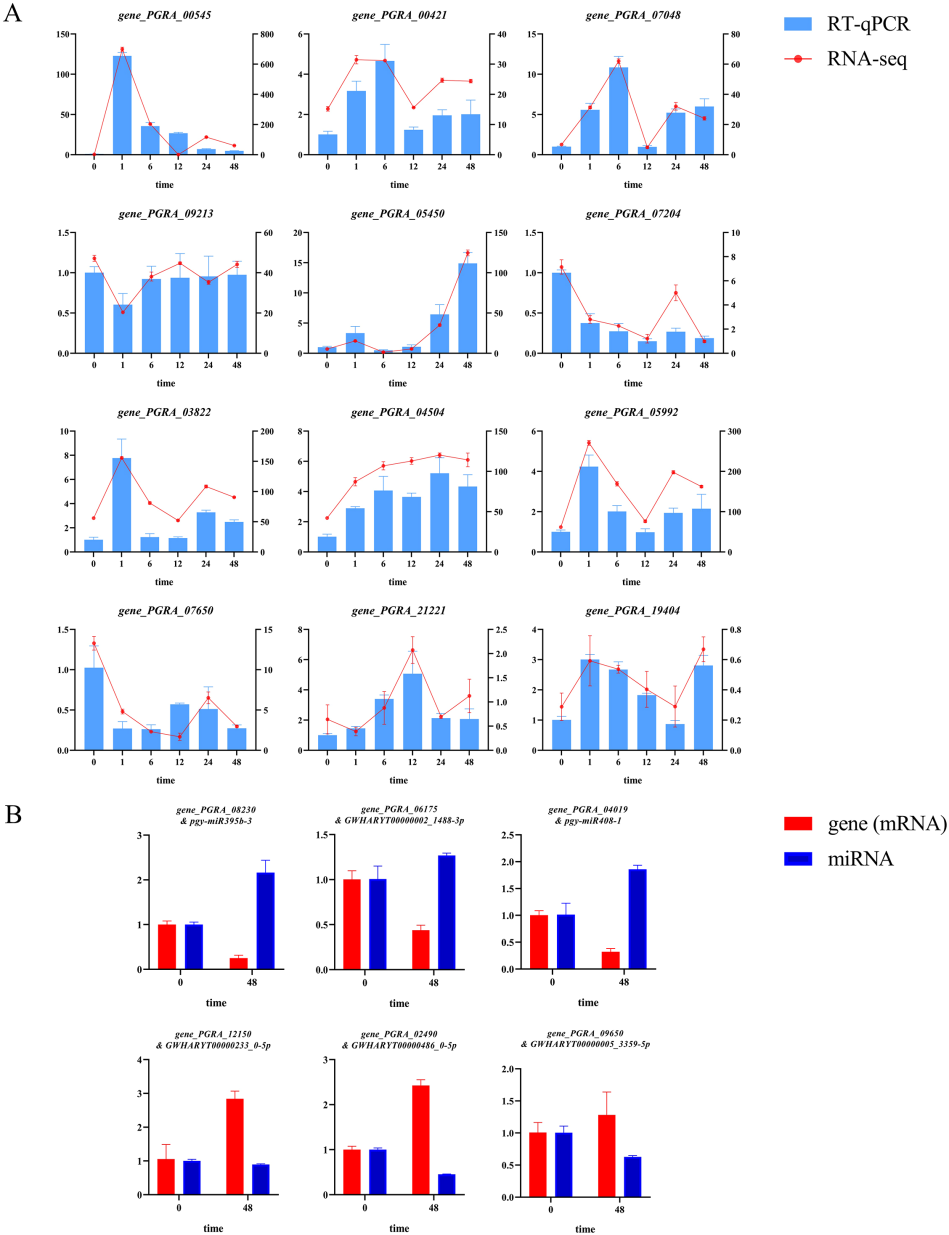
Validation of candidate differentially expressed genes and miRNA-mRNA regulatory pairs by qRT-PCR. **(A)** qRT-PCR validation of candidate differentially expressed genes. The left and right y-axes indicate the relative expression levels measured by qRT-PCR and RNA-seq, respectively. **(B)** qRT-PCR validation of miRNA-mRNA regulatory pairs. For each regulatory pair, the relative expression levels of the miRNA and its target gene are shown.

To assess the accuracy of the transcriptome sequencing results, validation was performed using qRT-PCR. Twelve differentially expressed genes were randomly selected from those identified in the integrated analysis for qRT-PCR detection. To further verify the reliability of the miRNA-mRNA regulatory network, an additional six miRNA-mRNA regulatory pairs were selected for qRT-PCR validation. These six pairs covered different expression patterns: three pairs exhibited up-regulated miRNA and down-regulated target gene (*pgy-miR395b-3* with its target gene PGRA_08230 (*BGLU11*), *GWHARYT00000002_1488-3p* with PGRA_06175, and *pgy-miR408–1* with PGRA_04019), and the other three pairs exhibited down-regulated miRNA and up-regulated target gene (*GWHARYT00000233_0-5p* with PGRA_12150, *GWHARYT00000486_0-5p* with PGRA_02490, and *GWHARYT00000005_3359-5p* with PGRA_09650). The results obtained by qRT-PCR were then compared with the sequencing data. The results showed that the expression trends of the vast majority of genes were highly consistent with the transcriptome sequencing data (**Figure 5A**). All six miRNA-mRNA pairs exhibited expression trends consistent with the sequencing data (**Figure 5B**). These experimental results further confirmed the good reliability of the transcriptome sequencing data obtained in this study.

### 3.8 Analysis of gene expression in the platycodin biosynthesis pathway

**Figure 6:**
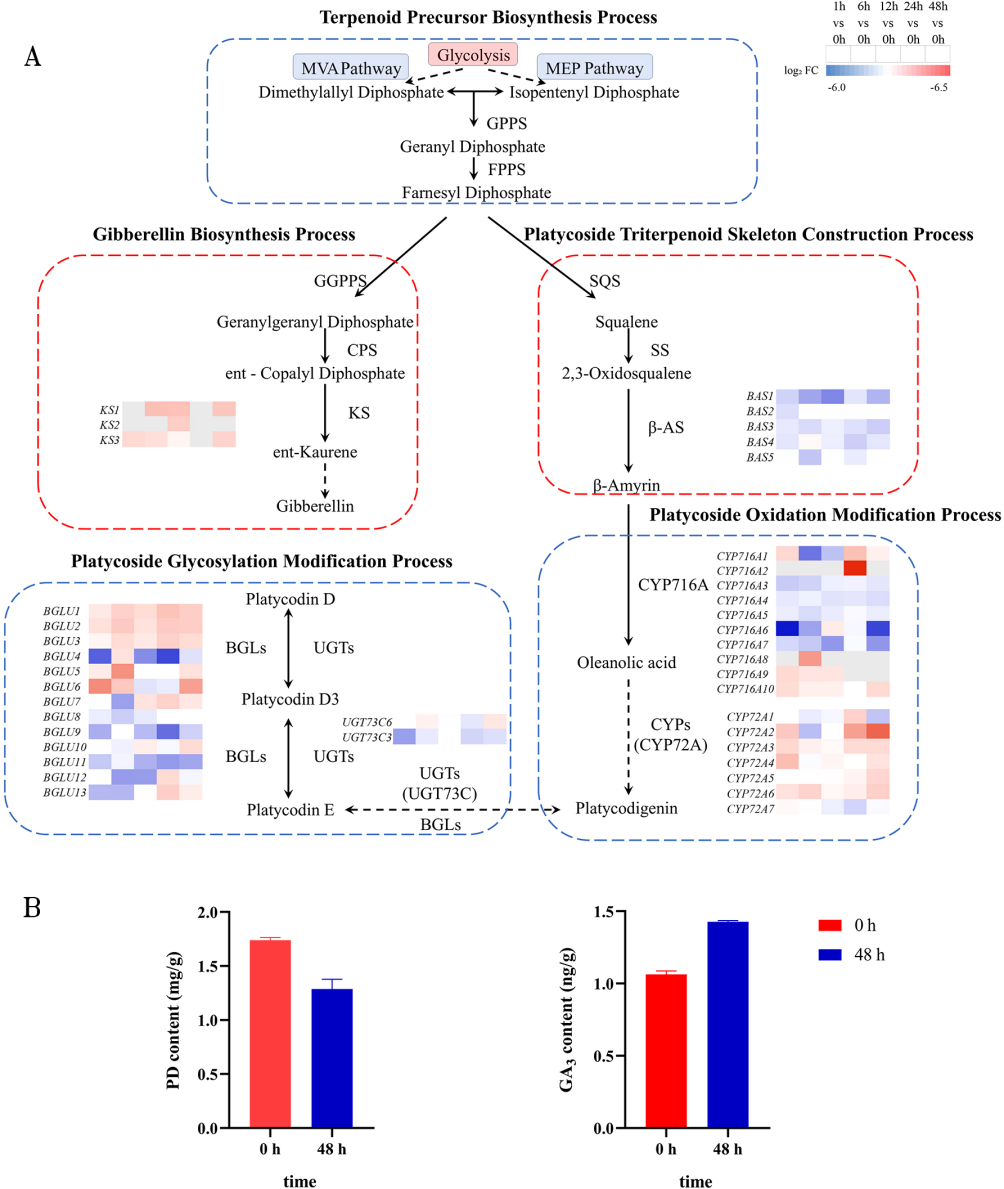
Gene expression and metabolite changes in the platycodin and gibberellin biosynthesis pathways under high-temperature stress. **(A)** Heatmap of annotated genes in the platycodin and gibberellin biosynthesis pathways. Identified enzymatic reactions are represented by solid arrows, whereas dashed arrows correspond to multi-step enzymatic processes. **(B)** Effects of 48 h high-temperature treatment on the contents of platycodin D and gibberellin GA_3_ in *P. grandiflorus* seedlings. Left panel: platycodin D content measured by UPLC; right panel: GA_3_ content measured by LC-MS. Data are presented as mean ± standard deviation (SD) (n = 3).

Triterpenoid saponins are the core secondary metabolites in *P. grandiflorus*, playing crucial roles in regulating osmotic balance, maintaining physiological homeostasis under stress conditions, and modulating growth and development (Zheng et al., 2021; Kundu et al., 2025). Previous metabolic pathway analysis indicated that high-temperature stress alters the expression of genes related to saponin metabolism in *P. grandiflorus*. As illustrated in the **Figure 6**, during the construction of the terpenoid backbone for platycodin biosynthesis, the expression levels of five differentially expressed genes annotated as β-amyrin synthase (β-AS), including *BAS1*, *BAS2*, *BAS3*, *BAS4*, and *BAS5*, were all down-regulated. The expression of *BAS2* and *BAS1* decreased significantly after 1 h of treatment, reaching their lowest levels at 1 h and 12 h post-treatment, respectively. The expression of *BAS4* and *BAS3* showed a fluctuating downward trend, attaining their lowest levels after 24h and 48 h of treatment, respectively. In contrast, *BAS5* reached its lowest expression level after 6 h of treatment.

Cytochrome P450 enzymes (CYPs) are core enzymes responsible for backbone modification in the platycodin biosynthesis pathway. In this study, 131 differentially expressed *CYP* genes were identified. Among them, 11-oxo-β-amyrin 30-oxidase and β-amyrin 28-monooxygenase have been specifically reported to be involved in triterpenoid saponin metabolism (Seki et al., 2011; Srivastava et al., 2024). During the oxidative modification stage of platycodin biosynthesis, the expression trends of key oxidase genes were analyzed. Among the seven differentially expressed genes annotated as 11-oxo-β-amyrin 30-oxidase (*CYP72A1*-*7*) and the ten annotated as β-amyrin 28-monooxygenase (*CYP716A1*-*10*), the expression levels of most family members increased with increasing stress duration. The expression of 11-oxo-β-amyrin 30-oxidase genes (including *CYP72A1*-*6*) was up-regulated, with *CYP72A2*, *CYP72A3*, *CYP72A5*, and *CYP72A6* reaching their peak expression levels at 48 h post-treatment, while the expression of *CYP72A7* was down-regulated. Among the ten β-amyrin 28-monooxygenase genes, except for *CYP716A3*, *CYP716A4*, *CYP716A5*, and *CYP716A7*, which were significantly down-regulated, the other six genes were up-regulated. It can be observed that under high-temperature stress, the saponin metabolic pathway exhibits a differential response pattern characterized by down-regulation of upstream genes and up-regulation of downstream genes. Similar patterns have been reported in other species. For example, in yeast (Chubukov et al., 2012), intermediate metabolites within the IMA architecture can lead to strong transcriptional activation of downstream genes and weak activation of upstream genes. This essentially represents a stress adaptation achieved by plants through metabolic flux redirection.

This study also identified 71 differentially expressed UDP-glucuronosyltransferase or UDP-glucosyltransferase (UGT) genes. According to previous reports, some members of the *UGT73* family have been confirmed to participate in the glycosylation modification of triterpenoid saponins (Augustin et al., 2012; Wei et al., 2025). During the glycosylation modification stage of platycodin biosynthesis, the expression patterns of two UDP-glucosyltransferase genes from the *UGT73C* family (*UGT73C6* and *UGT73C3*) were specifically analyzed. The expression of *UGT73C6* was significantly down-regulated after high-temperature treatment, with a slight recovery observed at 48 h post-treatment. The expression of *UGT73C3* was down-regulated, reaching its lowest level at 1 h after treatment initiation. Additionally, β-glucosidases (BGL) are also involved in glycosidic bond hydrolysis and the structural modification of saponin. Among the 13 differentially expressed genes annotated as β-glucosidase (*BGLU1-13*), the expression of 10 genes was significantly up-regulated, except for *BGLU8*, *BGLU9*, and *BGLU11*. The varied expression changes of these enzyme genes in response to high-temperature stress further confirm the close association between saponin biosynthesis and the HT stress response. Detailed gene annotation information is provided in **Supplementary Table 4**.

### 3.9 Analysis of DEMs and their target genes associated with the saponin metabolic pathway under high-temperature stress

**Figure 7:**
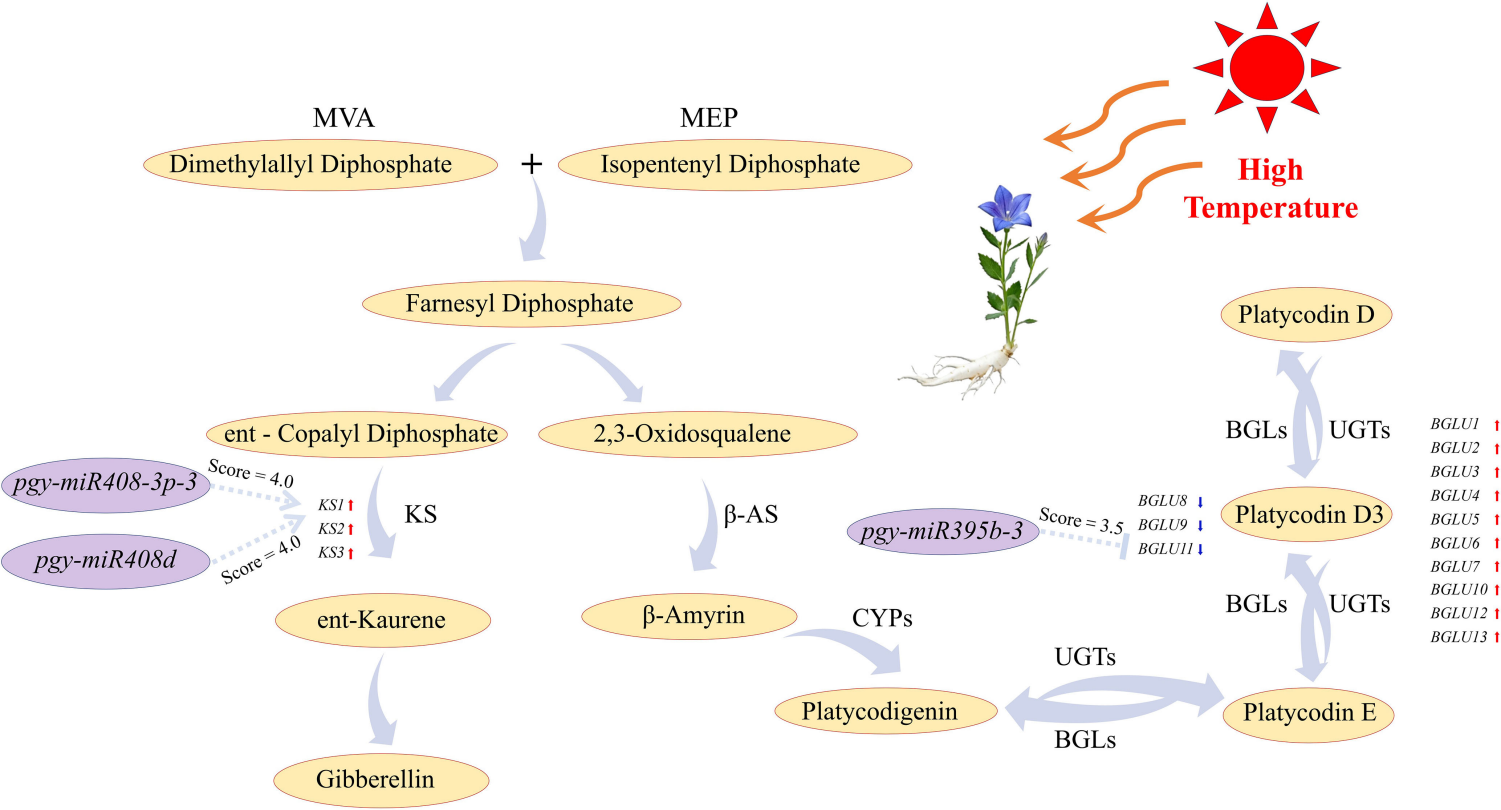
Expression relationships between DEGs and DEMs within the platycodin and gibberellin metabolic pathways under HT stress.

By analyzing the triterpenoid saponin metabolic pathway in *P. grandiflorus* under high-temperature stress, this study focused on the differentially expressed miRNAs and their corresponding differentially expressed target genes within this pathway. The integrated analysis identified potential regulatory nodes (**Figures 6A**, **7**). During the construction of the terpenoid backbone of platycodin biosynthesis, the expression of multiple genes encoding backbone synthesis enzymes (*BAS1-5*) was down-regulated. This pattern may reflect metabolic crosstalk or substrate competition between this pathway and other biosynthetic routes in *P. grandiflorus*, such that intermediate products are preferentially channeled into alternative downstream pathways to produce other stress-responsive metabolites. Notably, Gibberellin (GA), as a terpenoid hormone, shares the same precursors—isopentenyl diphosphate (IPP) and dimethylallyl diphosphate (DMAPP)—with platycodin biosynthesis. After IPP and DMAPP condense to form farnesyl diphosphate (FPP), the metabolic flux diverges. Therefore, a resource competition likely exists at the uppermost stream between the GA and platycodin biosynthetic pathways. Through integrated analysis, we found that the differentially expressed miRNAs *pgy-miR408-3p-3* and *pgy-miR408d* exert regulatory effects on enzyme genes within the GA metabolic pathway. Among the three DEGs annotated as ent-kaurene synthase whose expression was significantly up-regulated in response to HT stress, *KS1* was identified as a target of *pgy-miR408-3p-3* and *pgy-miR408d*. To further determine whether *pgy-miR408-3p-3* and *pgy-miR408d* co-target *KS1*, the precursor sequences of these two miRNAs were extracted, and global sequence alignment as well as secondary hairpin structure prediction were performed. The global alignment results from EMBOSS Needle (https://www.ebi.ac.uk/jdispatcher/psa) showed that the two precursor sequences shared only 47.9% identity and exhibited a marked difference in length (130 nt vs. 217 nt), indicating low precursor conservation (**Supplementary Figure 1**). Nevertheless, the mature miRNA regions were highly similar, with *pgy-miR408-3p-3* containing only an additional cytosine (C) at the 5’ end compared with *pgy-miR408d*, while the remaining bases were identical. Secondary structure prediction using the RNAfold web server (http://rna.tbi.univie.ac.at/cgi-bin/RNAWebSuite/RNAfold.cgi) further revealed that the secondary structures within the mature regions of the two miRNAs are also highly similar (**Supplementary Figure 2**). These results suggest that, despite the divergence in their precursors, the mature sequences of these two miRNAs are highly conserved, a characteristic feature of plant miRNA family members. Based on these observations, we hypothesize that *pgy-miR408-3p-3* and *pgy-miR408d* co-target *KS1* in a cooperative manner, although this conclusion requires further experimental validation.

In the glycosylation modification process of platycodin biosynthesis, UGTs facilitate the glycosylation of saponins, while β-glucosidases are involved in their deglycosylation. These two enzyme classes work coordinately to maintain the metabolic balance of saponins within *P. grandiflorus*. Modern research indicates that when plants are subjected to damage, they undergo deglycosylation modifications on saponins, resulting in lower-glycosylated saponins with higher biological activity that participate in defense responses (Lacchini et al., 2023). In this study, it was observed that under high-temperature stress, the expression of *UGT* genes involved in platycodin glycosylation was down-regulated, while the expression of several β-glucosidase genes was up-regulated. This aligns with previous scholarly findings that “stress induces saponin deglycosylation to rapidly produce defensive active molecules”. Notably, this study found that the expression of one specific β-glucosidase gene (*BGLU11*) was significantly down-regulated and negatively regulated by a differentially expressed miRNA (*pgy-miR395b-3*). This phenomenon suggests that, against the backdrop of overall activation of deglycosylation, plants may employ differential and precise regulation on distinct β-glucosidase genes.

In summary, under high-temperature stress, *P. grandiflorus* modulates its platycodin anabolic processes through a complex miRNA-mRNA regulatory network, exerting both indirect and direct effects. This coordinated regulation enables the plant to adapt to high-temperature environments and sustain homeostasis.

### 3.10 Changes in platycodin D and gibberellin GA_3_ contents under high-temperature stress

To investigate whether a metabolic flux reallocation from platycodin synthesis to the gibberellin pathway occurs in *P. grandiflorus* under high-temperature stress, the content of platycodin D was quantified in 0 h and 48 h samples using UPLC-ELSD, and the content of GA_3_ was measured using HPLC-MS.

As shown in **Figure 6B**, compared with the control group (0 h), the content of platycodin D in *P. grandiflorus* seedlings showed a decreasing trend after 48 h of high-temperature treatment, whereas the content of GA_3_ exhibited an increasing trend. Concurrently, transcriptomic data revealed down-regulation of upstream backbone synthesis genes (*BASs*) involved in saponin biosynthesis and up-regulation of the key rate-limiting enzyme gene (*KS*) in the gibberellin biosynthesis pathway. The consistent direction of changes between metabolites and transcriptome provides preliminary clues supporting the hypothesis that a carbon flux from platycodin synthesis toward the gibberellin pathway may occur under high-temperature stress.

## 4 Discussion

High temperature is a major limiting factor in the cultivation of *P. grandiflorus*. Therefore, elucidating the response mechanisms of *P. grandiflorus* to high-temperature stress is essential for developing heat-tolerant and high-yielding cultivars. However, research concerning the HT stress response in *P. grandiflorus* remains relatively limited. To Address the practical challenges that how extreme HT events associated with global warming affect *P. grandiflorus* production, this study systematically elucidated the molecular mechanisms underlying the response of *P. grandiflorus* seedlings to HT stress through integrated physiological, transcriptomic, and miRNAomic analyses.

Previous studies have reported that high-temperature stress can activate the plant antioxidant system, and as the stress duration prolongs, the activities of antioxidant enzymes within this system increase (Ahmad et al., 2010; Hong et al., 2023; Mishra et al., 2024). The results of this study are consistent with these findings: the activities of SOD, POD, and APX exhibited a fluctuating upward trend following high-temperature treatment. The activities of SOD and POD peaked at 48 h, while APX activity reached its peak at 24 h. MDA is a hallmark product used to assess the extent of membrane lipid peroxidation in plant cells (Shi et al., 2012). The MDA is widely used as a key marker of cellular injury because its accumulation reflects the extent of membrane lipid peroxidation and, consequently, the degree of structural damage to cell membranes (Montero-Palmero et al., 2014). H_2_O_2_ is a key reactive oxygen species in plants. Under normal physiological conditions, H_2_O_2_ levels are maintained at a low concentration and are promptly scavenged by the antioxidant system. However, under stress conditions, the rate of ROS generation can markedly surpass the intrinsic scavenging capacity of the antioxidant defense system, thereby leading to a pronounced accumulation of H_2_O_2_ within plant tissues (Quan et al., 2008). Excessive H_2_O_2_ can directly damage biomacromolecules such as nucleic acids, proteins, and membrane lipids (Alam et al., 2025), inducing oxidative damage (Černý et al., 2018). Therefore, H_2_O_2_ is commonly used as a key indicator to evaluate ROS accumulation and the degree of oxidative stress in plants. In this study, the contents of MDA and H_2_O_2_ initially increased and subsequently decreased as the stress duration extended. These results demonstrate an adaptive response of *P. grandiflorus* to high-temperature stress. The initial accumulation of both MDA and H_2_O_2_ was triggered by an imbalance in ROS metabolism at the early stage of stress. Subsequently, as the plant activated its antioxidant system to eliminate excess H_2_O_2_, the process of membrane lipid peroxidation was suppressed, leading to a subsequent decline in MDA content.

Transcriptome sequencing analysis revealed that high-temperature treatment for 1 h, 6 h, 12 h, 24 h, and 48 h, resulted in the identification of, 4,996, 5,693, 4,358, 5,130, and 5,895 differentially expressed genes were identified, respectively. Both GO and KEGG enrichment analyses revealed that the differentially expressed genes were predominantly associated with pathways related to secondary metabolism. Specifically, the results highlighted significant enrichment in secondary metabolic processes and the biosynthesis of secondary metabolites. The seedling stage represents the developmental phase during which plants are most sensitive to stress, and extensive transcriptional reprogramming at this stage forms the basis for activating comprehensive defense mechanisms. In this study, the identification of thousands of differentially expressed genes under high-temperature stress aligns with the scale of the heat stress response observed in watermelon (*Citrullus lanatus* L.) (Song et al., 2020) or quinoa (*Chenopodium quinoa* Willd.) (Xie et al., 2023) seedlings, collectively supporting the prevalent strategy whereby seedlings mobilize a vast array of genetic resources to counteract stress. miRNA sequencing results showed most miRNAs were 21–24 nt in length, consistent with previous findings (Ma and Hu, 2023) and has also been reported in cotton (*Gossypium hirsutum* L.) (Zhao et al., 2019), *A. thaliana* (Rajagopalan et al., 2006), and rice (*O. sativa*) (Sunkar et al., 2008). In this study, a total of 62 differentially expressed miRNAs were identified, corresponding to 32 differentially expressed target genes, resulting in 58 miRNA-mRNA regulatory pairs.

Among these miRNA-mRNA pairs, the ent-kaurene synthase gene is regulated by members of the differentially expressed *pgy-miR408-3p-3* and *pgy-miR408d*, whereas, the β-glucosidase gene was a target of the differentially expressed miRNA *pgy-miR395b-3*. The former act as core rate-limiting enzyme in the early stages of gibberellin biosynthesis, while the latter is involved in the late-stage glycosylation modifications of platycodins. As an important class of plant hormones, all of these GAs play a significant regulatory role in mediating plant responses to high-temperature stress. For instance, studies have shown that GA can significantly increase the activities of antioxidant enzymes such as superoxide dismutase and peroxidase (Khan et al., 2020; Guo et al., 2022) and promote the accumulation of non-enzymatic antioxidants such as glutathione and proline (Zuo et al., 2022; Zhu et al., 2025), thereby facilitating the scavenging of excess intracellular reactive oxygen species and alleviating oxidative damage caused by extreme temperatures. Notably, plant response to high-temperature stress is mediated by a multilayered, synergistic network. In addition to the GA-mediated antioxidant system that scavenges ROS and maintains cellular homeostasis, dynamic modifications of secondary metabolites are also considered as a key strategy for resistance to both biotic and abiotic stresses. When plants are exposed to stress or sustain cellular structural damage, deglycosylation can convert highly glycosylated, storage-form saponins into more active, less glycosylated forms that actively participate in defense responses (Lacchini et al., 2023). The central executor of this process is β-glucosidase, which belongs to the glycoside hydrolase family. This enzyme specifically recognizes and hydrolyzes glycosidic bonds, catalyzing the removal of glucose residues from highly-glycosylated saponins (Lacchini et al., 2023) while simultaneously releasing free monosaccharides into the cellular metabolic pool (Singh et al., 2016). This process not only supplements carbon sources for plants under stress, providing raw materials for other carbohydrate metabolism pathways, but also elevates the intracellular levels of soluble sugars such as glucose and sucrose, thereby helping to cellular osmotic balance and membrane stability (Krasensky and Jonak, 2012). These intricate regulatory networks, by mediating the metabolic synthesis in *P. grandiflorus*, ultimately enhance the plant’s adaptability to high-temperature environments.

Compared to previous studies that primarily focused on physiological responses or single-omics data, the strength of this work lies in its adoption of a multi-omics integrative strategy. This study is the first time in *P. grandiflorus* to establish a preliminary regulator network for platycodin metabolism under high-temperature stress, spanning from transcriptional to post-transcriptional levels. Furthermore, this study extended the analysis to explore its connection with the gibberellin biosynthesis pathway. These findings constitute valuable molecular and genetic resources for this non-model plant. Unlike model plants such as *Arabidopsis* and rice (*O. sativa*), in which research on high-temperature stress has primarily focused on universal thermotolerance mechanisms involving heat shock proteins and signal transduction, *P. grandiflorus*, as a medicinal and edible plant, exhibits a unique heat stress response characterized by the dynamic reprogramming of secondary metabolism, particularly triterpenoid saponins. In this study, the saponin biosynthesis pathway in *P. grandiflorus* under high-temperature stress displayed a distinct differential expression pattern, with down-regulation of upstream backbone synthesis genes (*BASs*) and up-regulation of most downstream oxidative modification genes (*CYP72A/CYP716A*). This pattern shows both similarities to and species-specific differences from reports in *Panax notoginseng*, where water stress induced up-regulation of saponin synthase genes (Liao et al., 2017), and in *Astragalus membranaceus*, where drought stress down-regulated saponin-related genes (Ma et al., 2026). Under high-temperature stress, *P. grandiflorus* does not simply up-regulate or down-regulate the entire saponin pathway; instead, it likely redirects carbon skeletons from saponin synthesis toward hormone pathways such as gibberellin via metabolic flux reallocation, while rapidly producing defense-active, low-glycosylated saponins through the dynamic balance of glycosylation and deglycosylation. This may represent a distinctive molecular feature of *P. grandiflorus* in adapting to high-temperature stress, differentiating it from other medicinal plants.

It should be noted that miRNA sequencing in this study was performed only at two time points (0 h and 48 h). Although this design effectively captures the miRNA regulatory changes associated with metabolic reprogramming in the late phase of high-temperature stress, it fails to reflect the dynamic responses of miRNAs during the early phase of stress. Therefore, we recommend that future studies incorporate additional early time points (e.g., 12 h and 24 h) for miRNA sequencing. Furthermore, this study found that *pgy-miR408-3p-3* and *pgy-miR408d* were associated with the up-regulated expression of the *KS1* gene. Further sequence analysis revealed that, although the precursor sequences and secondary structures of these two miRNAs differ substantially, their mature sequences are highly similar, suggesting that they may co-target *KS1* as members of the *miR408* family. Therefore, we hypothesize that these two miRNAs co-target *KS1* in a cooperative manner rather than via cross-targeting. Since direct experimental evidence from degradome sequencing or transient co-expression assays is currently lacking, this conclusion requires further experimental validation. In addition, the high-temperature treatment used in this study was acute (40 °C for 48 h). Although this effectively simulates the impact of extreme heat events on seedlings, it may not fully reflect field conditions with diurnal fluctuations and long-term, progressive heat stress. Therefore, the conclusions are primarily applicable to explaining the early response mechanism of *P. grandiflorus* to acute short-term high-temperature stress. Moreover, the samples analyzed in this study were whole seedlings, without distinguishing among roots, stems, and leaves. Given that the medicinal part of *P. grandiflorus* is the root and that saponin synthesis exhibits tissue specificity (with the root being the primary organ for saponin accumulation), the metabolic regulatory networks under high-temperature stress may differ significantly between roots and above-ground parts. Future studies should combine stable isotope tracing (e.g., ¹³C-labeled glucose or CO_2_) to dynamically track the flux of carbon skeletons among triterpenoid saponin, gibberellin, and other terpenoid pathways, together with tissue-specific expression analysis, to more precisely elucidate the spatiotemporal dynamics of metabolic reallocation. Meanwhile, this research is still in the early stages of investigating the underlying mechanism. The main conclusions are based on correlation-based analyses, lacking direct experimental validation of the functions of key candidate genes and miRNAs. Moving forward, future research should focus on functional validation, employing approaches such as genetic transformation to confirm the roles of core regulatory components. Additionally, integrating data from proteomics and metabolomics data would be essential to construct a complete regulatory cascade from genes to phenotypes. Ultimately, bridging laboratory findings with practical production will facilitate the application of stress-resistance breeding, thereby providing substantive theoretical support and solutions for the stable development of the *P. grandiflorus* industry.

## Data Availability

The RNA-Seq and miRNA-Seq data in this study have been deposited in the NCBI Sequence Read Archive (SRA) under the BioProject accession number PRJNA1331435 and PRJNA1331495.
