# Supplementary material for: Transcriptome and miRNAome analysis reveals expression profiles of platycodin biosynthesis-related genes and their potential miRNA regulators in Platycodon grandiflorus under high-temperature stress
Source: Front Plant Sci. 2026 May 22;17:1820112. doi: 10.3389/fpls.2026.1820112 (PMC13236508; doi:10.3389/fpls.2026.1820112)

Calibration Curve Report

Analyte Name: GA3-1

Acquisition Time: 04/10/2026 04:22:59 AM

Analyte Type: Quantifier

Project: Default Project

LC Method: LC_zwjs_0.02FA+ METH_40_10µL_15min

Injection Volume (µL): 10

Instrument Model: QSight 420

MS Method: 1_MS_zwjs30+cms4+ is6_fenduan-15min-2500-1

Result File: 2026.04.10-ZWJS-adj

Reporting Time: 04/10/2026 05:33:09 PM

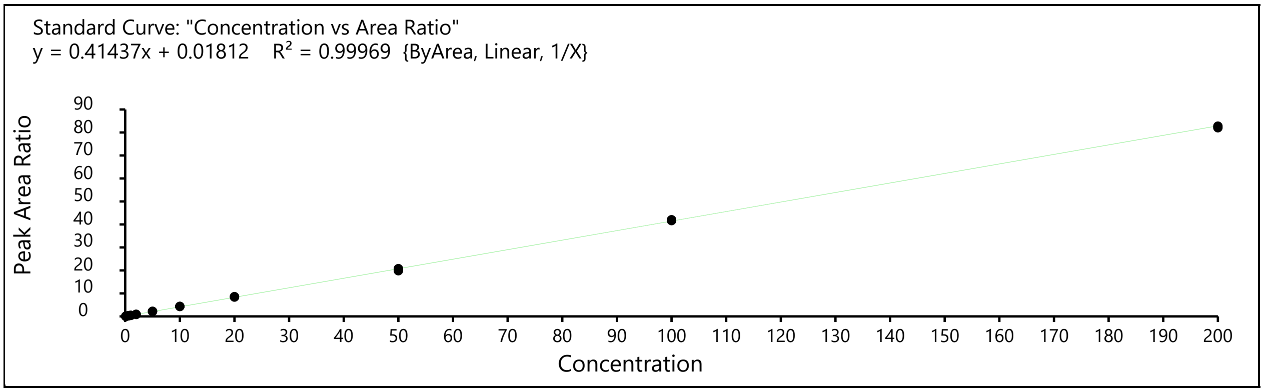

Supplement: Supplementary file 1 [file DataSheet1.zip › Supplementary Files/PD&GA data/GA-Standard Curve.pdf]
