# Supplementary figures and images for: Transcriptome and miRNAome analysis reveals expression profiles of platycodin biosynthesis-related genes and their potential miRNA regulators in Platycodon grandiflorus under high-temperature stress
Source: Front Plant Sci. 2026 May 22;17:1820112. doi: 10.3389/fpls.2026.1820112 (PMC13236508; doi:10.3389/fpls.2026.1820112)

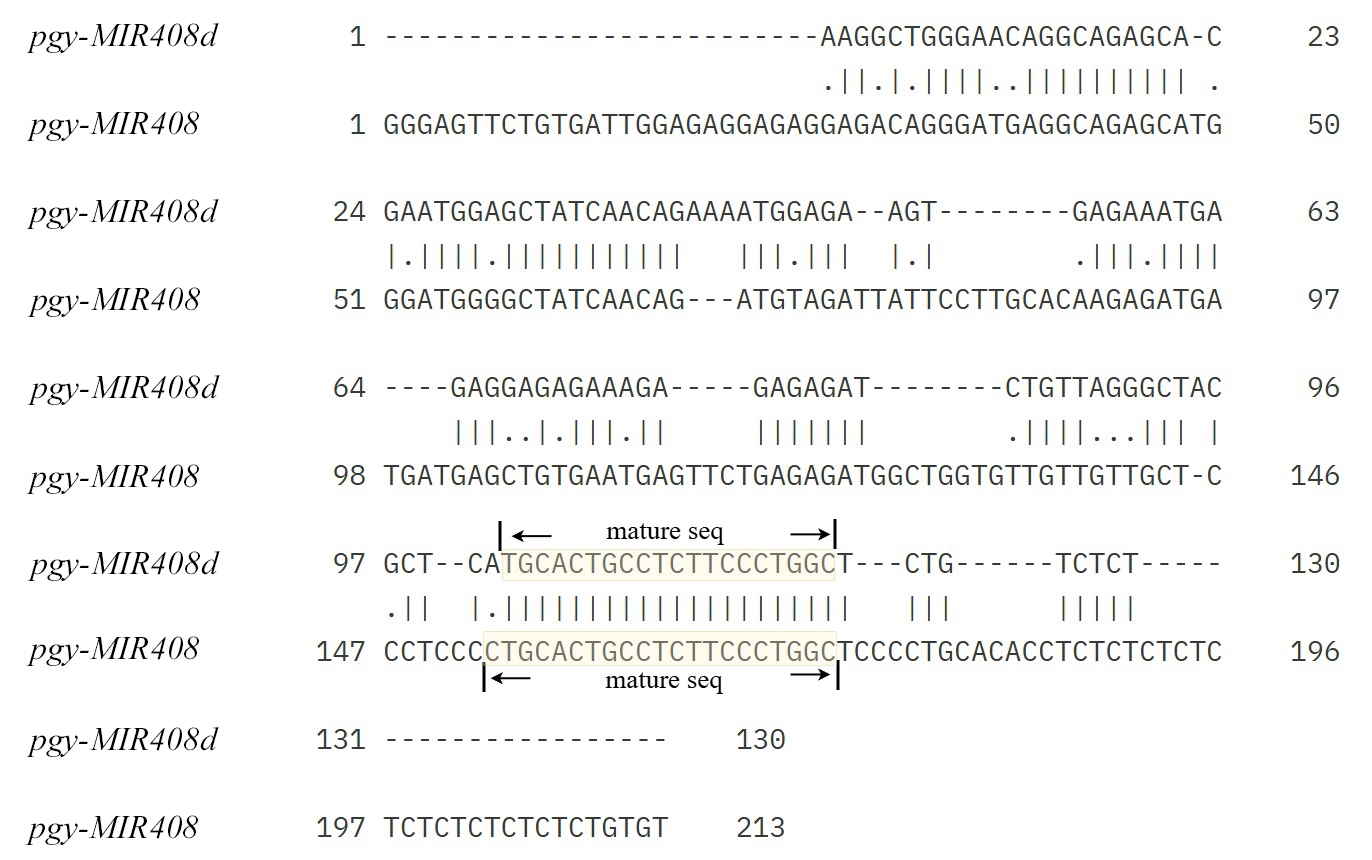

Supplement: Supplementary file 1 [file DataSheet1.zip › Supplementary Files/Figure S1.jpg]

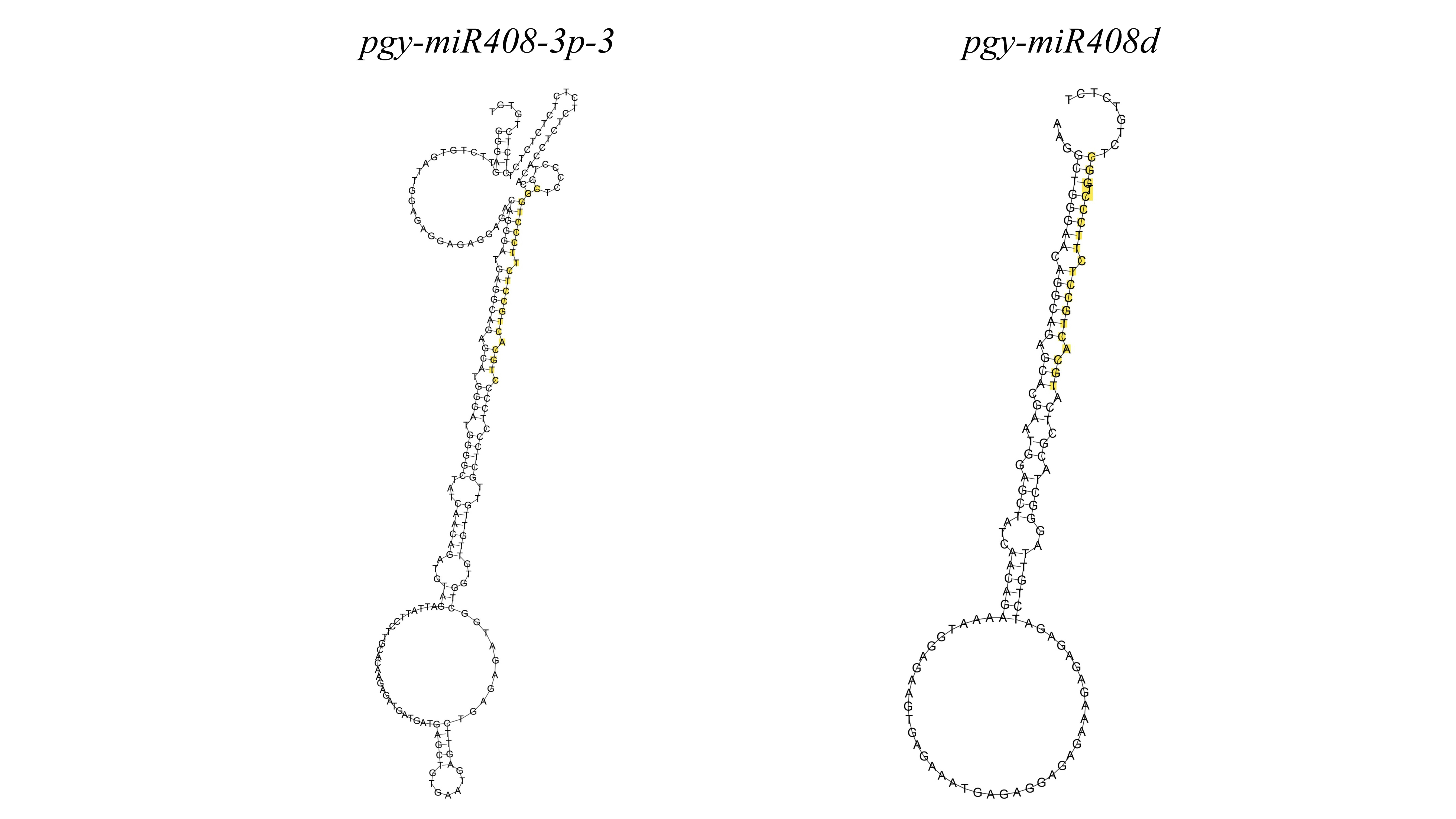

Supplement: Supplementary file 1 [file DataSheet1.zip › Supplementary Files/Figure S2.jpg]
